# A comparison of acceptability of contraceptive vaginal rings, pills, and injectables among cisgender women in Kenya and Zimbabwe: protocol for a mixed-methods study

**DOI:** 10.12688/gatesopenres.16315.1

**Published:** 2025-03-03

**Authors:** Chelsea B. Polis, Francis O. Obare, Irene V. Bruce, Cynthia Banda, Lisa B. Haddad, Antwanette Heyns, Petros Isaakidis, Mercy Kamupira, Terrance Kufakunesu, Zachary A. Kwena, Farai Machinga, Regina F. Magore, Aleck Mapangire, Mercy Marimirofa, Matheus Mathipa, Sanyukta Mathur, Mary Mudavanhu, Tatenda P. Mujuru, Prisca Mutero, Betty Njoroge, Collen Nyatsambo, Sarah Okumu, Leah Omondi, Tevyne Omondi, Marlena G. Plagianos, Greshon Rota, Samuel Sithole, Bruce Variano, J. Brady Burnett-Zieman, Petina Musara, George Odwe, Gerald Hangaika, Serah Gitome, Elizabeth A. Bukusi, Kuziwa Kuwenyi

**Affiliations:** 1 Population Council, New York, New York, 10065, USA; 2 Population Council Kenya, Nairobi, Nairobi County, Kenya; 3 Médecins Sans Frontières, Harare, Zimbabwe; 4 IPM South Africa NPC, Population Council, Johannesburg, South Africa; 5 Médecins Sans Frontières, Cape Town, South Africa; 6 Harare Health Research Consortium, Harare, Zimbabwe; 7 Kenya Medical Research Institute, Nairobi, Nairobi County, Kenya; 8 Zimbabwe National Family Planning Council, Harare, Zimbabwe; 9 Population Council, Washington, DC, USA; 10 City of Harare, Health Services,, Harare, Zimbabwe; 11 University of Washington, Seattle, Washington, USA; 12 University of California San Francisco, San Francisco, California, USA

**Keywords:** Acceptability, satisfaction, intravaginal rings, contraceptive pills, injectables, mixed-methods, Kenya, Zimbabwe

## Abstract

**Background:**

Expanding contraceptive options could better meet users’ diverse needs and preferences. Annovera ^®^ is a contraceptive vaginal ring that provides a year of pregnancy prevention while remaining under user control and allowing for regular menstrual cycles. This method may also help to reduce burdens on some health care and supply chain systems. However, knowledge gaps exist regarding initial and ongoing acceptability of contraceptive vaginal rings in African settings.

**Methods:**

We will undertake an open-label, non-randomized, two-arm, parallel clinical acceptability study with an embedded qualitative component, based in clinics providing contraceptive services in Kenya and Zimbabwe. Women aged 18-45 interested in newly initiating or switching contraception will choose from among all available contraceptive options, including Annovera. We aim to enroll 200 participants selecting Annovera and 200 participants selecting either contraceptive injectables or pills. We will compare method uptake, continuation, and satisfaction over one year. Participants will complete questionnaires administered by study staff during two in-person visits (a screening/enrollment visit, and an end of study visit after 52 weeks of method use or at discontinuation) and four phone appointments (at 4, 12, 24, and 36 weeks of use). We will evaluate used rings for discoloration and residual drug levels. The qualitative component involve in-depth interviews with women in the clinical study, their sexual partners, and their service providers, to further examine drivers of and barriers to interest in and use of contraceptive vaginal rings.

**Discussion:**

This study will explore acceptability of contraceptive vaginal rings in ‘real-world’ contraceptive service settings in two African countries. Findings will be based on actual ring use and contextualized via comparison to two other commonly available methods. As vaginal rings are being considered for multiple reproductive health indications, this work can fill key knowledge gaps and empower decision-makers with information needed to inform future investments in reproductive health.

## 1.0 Introduction

Globally, about 48% of all pregnancies are unintended, corresponding to about 121 million unintended pregnancies annually. ^1^ In low- and middle-income countries (LMICs), nearly 218 million women (24% of all women who want to avoid a pregnancy) have an unmet need for modern contraception, ^2^ and this unmet need is highest in Africa. Meeting this need in LMICs could prevent 76 million unintended pregnancies, 21 million unplanned births, 26 million unsafe abortions, and 70,000 maternal deaths. ^2^ Expanding method choice could better address the needs and reproductive intentions of more people, increase overall contraceptive uptake, continuation, and satisfaction, and reduce unintended pregnancies. ^3, 4^

The current array of contraceptive options available in LMICs could be expanded to better meet diverse needs and preferences of users. For example, equitable access to long-acting reversible contraceptive (LARC) methods is constrained by gaps in the availability of trained providers, lack of regular access to commodities, concerns about access to removal, and interference with regular menstrual bleeding. ^5– 11^ These challenges highlight the need for user-controlled methods to support contraceptive autonomy and enable self-care. A safe, effective, easy-to-use, long-acting contraceptive option under the control of the user, particularly one which maintains regular bleeding patterns, could be a useful addition to the method mix in LMICs. Furthermore, as shown by disruptions to contraceptive service provision and supply chains during the COVID-19 pandemic, ^12^ self-administered contraceptive options could reduce reliance on healthcare systems, mitigate access disruptions, and increase individual reproductive autonomy. ^13^

The contraceptive registered in the United States as Annovera ^®^ (manufactured by Sever Pharma Solutions/QPharma, Malmö, Sweden) fits the self-care model designed to meet women’s contraceptive needs, allows for regular menstrual cycles, and expands method choice. It is a flexible, silicone elastomer vaginal contraceptive ring system containing segesterone acetate (103 mg) and ethinyl estradiol (17.4 mg), which was developed by the Population Council and approved by the US Food and Drug Administration (FDA) in August 2018. ^14^ Annovera has an acceptable safety profile and contraceptive efficacy similar to other combined hormonal contraceptive methods. ^15, 16^ A single ring provides one full year (thirteen 28-day cycles) of pregnancy prevention, remains under the user’s control (i.e., can be stopped without consulting a healthcare provider), and does not require daily user action. The ring is inserted vaginally by the user for a 21-day interval, followed by a 7-day ring free interval, and can then be reinserted for a subsequent use cycle. In clinical trials, the typical use Pearl Index pregnancy rate was approximately 3%. ^16^ Annovera does not require refrigeration and may reduce the burden on some health care and supply chain systems as compared with provider-dependent methods (IUDs, implants) since it is procedure-free, and can provide a longer duration of protection (one year) than some other self-administered products (e.g., oral contraceptives, Sayana ^®^ Press). Two Phase III trials on Annovera enrolled over 2,300 participants from the United States, Europe, Australia, Brazil, Chile, and the Dominican Republic, but did not include sites in Africa.

Contraceptive vaginal rings can deliver one or more drugs into the vaginal environment. These drugs can act locally or systemically, with steady release over a period of time for non-daily continuous protection, potentially fewer side effects, and less frequent administration requirements as compared with oral routes. ^17^ They have emerged as a prominent delivery formulation for development of products targeting an array of indications, such as contraception (e.g., Annovera, NuvaRing), HIV prevention (e.g., Dapivirine vaginal ring), menopause management (e.g., Estring, Femring, Fertiring), and in the ongoing development of multipurpose prevention technologies to simultaneously prevent unintended pregnancy, HIV and/or other sexually transmitted infections (STIs). ^18^

Despite the potential advantages of contraceptive vaginal rings in LMICs, some studies have prompted skepticism about the acceptability of vaginal rings in these contexts. In two studies asking potential users about their *hypothetical* willingness to try various delivery forms, rings did not perform as favorably as other forms. ^19, 20^ A qualitative data review suggested that women may have initial concerns about using vaginal rings (e.g., related to insertion or removal, cleanliness, or discomfort), but that these concerns are typically overcome with time during actual ring use. ^21^ Indeed, systematic reviews on vaginal ring acceptability in LMICs in which women were able to actually try using rings suggest that, across indications and geographic areas, vaginal rings have favorable acceptability which tends to increase as women gain actual use experience. ^22, 23^ Another literature review suggested that few women were concerned about vaginal insertion, noting that “some reproductive health experts may have overestimated concerns about vaginal insertion and its impact on the demand for these contraception methods”. ^24^ In one randomized cross-over study of three non-medicated methods (daily oral tablets, monthly injections, and a monthly vaginal ring), women rated their experience of using the ring more positively than using pills, and the ring also had the greatest increase in favorability over time. ^25^

To address knowledge gaps regarding initial and ongoing acceptability of contraceptive vaginal rings in African settings, we will undertake the Contraceptive Acceptability REsearch (CARE) study.

## 2.0 Protocol

We followed SPIRIT reporting guidelines in developing this protocol manuscript. ^26^

*https://doi.org/10.7910/DVN/ZFJZ2D*

### 2.1 Study design

CARE is an open-label, non-randomized, two-arm, parallel clinical acceptability study with an embedded qualitative component, based in clinics providing contraceptive services in Kenya and Zimbabwe. In the clinical study (hereafter, “CARE-clinical”) women seeking to newly initiate or switch to a different method of contraception at one of these sites will be offered balanced contraceptive counseling on all available methods. Annovera will be presented as one option among all other options available at that site, all of which are offered free-of-charge. Women will be invited to choose the method with which they feel most comfortable. All women who wish to try Annovera, and a subset of women who wish to try either oral contraceptive pills (OCs) or depot medroxyprogesterone acetate (DMPA) injections, will be provided with information on the study, and if interested, will undergo informed consent and eligibility screening. Since Annovera is not registered in the study countries, only study participants will be eligible to use the ring. If study participation is declined, all other contraceptive options will remain available to that individual. CARE-clinical will assess comparative method uptake, continuation, and satisfaction over the course of one year, comparing women who choose Annovera versus those who choose OCs or DMPA. We selected OCs and DMPA as comparators because they are the most widely used non-LARC methods in both study countries, and thus are the most likely methods chosen by people who may otherwise try Annovera. In addition, unlike IUDs or implants, all three methods require some degree of user action for continued protection, making them more comparable.

All enrolled participants will complete two in-person visits (a screening/enrollment visit, and an end-of-study visit, occurring either after 52 weeks of method use or at the point of early discontinuation), and four phone appointments (at 4, 12, 24, and 36 weeks of use). Study questionnaires will be administered at each of these six appointments. CARE-clinical will adhere to Good Clinical Practice (GCP) guidelines for clinical studies.

The qualitative component (hereafter, “CARE-qualitative”) will assess factors influencing acceptability and perceptions of Annovera from women in both arms of CARE-clinical, and perceptions about Annovera from both the male sexual partners of women using Annovera and from healthcare providers offering Annovera. We will achieve this via in-depth interviews (IDIs) with four groups at different points in time:

• women currently using Annovera or those who tried and discontinued Annovera (serial cross-sectional interviews occurring at 4, 24 and 52 weeks after method initiation),
• male partners of women using Annovera (interviewed once between 12-24 weeks after Annovera initiation),
• women who do not choose Annovera during contraceptive counseling and instead choose OCs or DMPA (interviewed once between 4-8 weeks after contraceptive selection), and
• health care staff from the participating clinics providing Annovera (interviewed once towards study end, when most participants have completed 13 months or discontinued method use).

As the vaginal ring is a novel delivery system for contraception in the proposed settings, we will also undertake various community engagement activities to help ensure that potential end-users, advocates, community leaders, and other key stakeholders understand the purpose of these studies, have accurate information about all methods, and have access to the study findings.

### 2.2 Study settings and populations

CARE-clinical will recruit potential participants from non-pregnant women seeking contraception from standard service provision sites in Kibera (Nairobi), Kenya and Mbare (Harare), Zimbabwe. The Zimbabwe site is an adolescent sexual and reproductive health project run by Médecins Sans Frontières (MSF) which focuses on adolescents and young people aged 10-24. The Kenya site is a government facility that serves all ages. Participants will be aged 18-24 in the Zimbabwe site and 18-45 in the Kenya site. Across both sites, we aim to recruit approximately 200 Annovera users and 200 OC/DMPA users.

CARE-qualitative will recruit participants from the Annovera arm of CARE-clinical (up to 30 women in the Kenya site and 18 in the Zimbabwe site) for serial cross-sectional IDIs with women who are using or who have discontinued using Annovera. CARE-qualitative will also recruit potential participants from the OC/DMPA arm of CARE-clinical (up to 6 in each study site) for a one-time IDI focused on reasons for method selection. We will also recruit male partners of Annovera-using women (up to 6 male partners in each study site) and health care staff from the participating clinics providing Annovera (up to 3 staff members per site).

### 2.3 Study products and provision

Annovera (segesterone acetate and ethinyl estradiol vaginal system) is a silicone elastomer, nonbiodegradable, flexible, opaque white, vaginal ring containing 103 mg segesterone acetate (SA) and 17.4 mg ethinyl estradiol (EE). Annovera is designed to be used for up to 13 cycles (1 year), with each cycle of use consisting of 28 days (21 days with the ring in the vagina, followed by 7 days with the ring removed). Annovera is 56 mm in overall diameter and 8.4 mm in cross-sectional diameter. A black compact carrying case will be provided with Annovera for storage of the ring by participants during each 7-day out interval. Each Annovera-selecting participant will have the opportunity to use their ring for 52 weeks (13 cycles, kept in for 3 weeks and removed for 1 week each cycle), or until they choose to discontinue the method or study. Women who enroll in the Annovera arm of CARE will be given a paper-based wheel tool, designed for this study, that can help them track upcoming insertion and removal dates.

Clinical study staff will instruct participants who choose to try Annovera on the correct use of the ring, including the importance of continuous ring use during the 21-day treatment cycles. They will also instruct users to reinsert the ring immediately if it is expelled or removed, and to ensure that the ring is not out of the participant’s vagina for more than two hours (cumulatively) during the entire 21-day treatment cycle. Participants will be informed that if they lose their Annovera ring or it becomes damaged, this should be immediately reported to the study site, and that the study staff will provide up to one replacement ring, if supplies are available. If no replacement Annovera ring is available, the participant will be discontinued from the study.

We developed contextually appropriate counseling materials, to help ensure that providers can offer unbiased, balanced counseling across all contraceptive methods already available at their site, in addition to supporting them in offering Annovera as a new method in their context. In conjunction with study sites, we developed an array of patient-facing materials to support contraceptive counseling and recruitment procedures. This includes flyers about the CARE study and about Annovera, a three-part flipbook (containing information about all contraceptive methods available at the clinic, detailed information on Annovera, and general information about participation in CARE), and a video describing Annovera and the CARE study. Study staff will also provide demonstrations about inserting and removing the ring by using a placebo ring and a translucent plastic pelvic model.

Women who choose to try Annovera will be compared with women opting to initiate OCs (either combined or progestin-only) or DMPA; these methods will be dispensed according to existing standards of care in each setting. All study participants will receive an SMS text message one week after product initiation, with a reminder that they may contact the study site if they have any questions or concerns with their selected contraceptive method.

### 2.4 Study procedures

*2.4.1 Recruitment*

**Figure 1.**
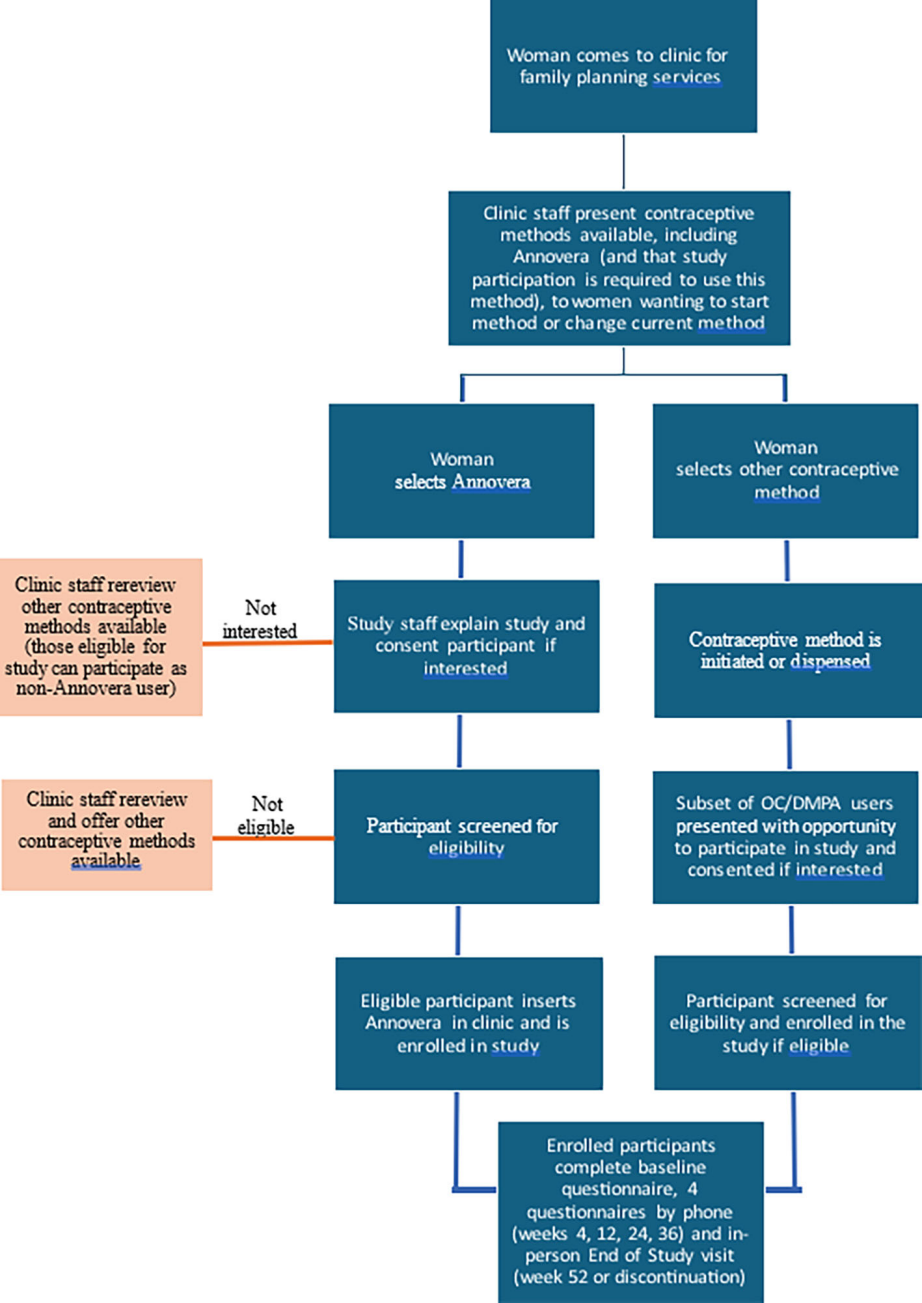
CARE-clinical study design schematic.

CARE-clinical : Recruitment will primarily take place in the contraceptive service provision sites. Women seeking to newly initiate or switch to a different method of contraception at these sites will receive information about the various contraceptive options available, including Annovera. Those expressing interest in participating in CARE-clinical will undergo a recruitment and screening process as described in Figure 1.

Each of the two sites will aim to recruit 200 women total; 100 in each of the two study arms (Annovera and OC/DMPA). As OCs and DMPA are commonly used in these contexts, enrollment into the OC/DMPA arm may proceed more quickly. To avoid the potential impacts of differential pacing of enrollment by arm, the rate at which we invite those who choose OCs or DMPA to participate in the study will be informed by the rate at which women self-select to enroll in the Annovera arm.

In addition to recruitment in the clinics, outreach teams will provide general contraceptive and sexual and reproductive health education activities within the community (e.g., salons, marketplaces, venues of commemorative events, etc.). Women who indicate interest in CARE during these outreach sessions will be pre-screened by site staff using a simplified version of the study inclusion criteria to determine presumptive eligibility. Potential participants will then be invited to the clinic for a screening visit. Participants who wish to involve any other individual (e.g., family member or current sexual partner) in their decision on whether to participate will be encouraged to bring that person to the clinic, where a study staff member can explain the study and answer questions.

CARE-qualitative *:* We will ask women in the Annovera arm of CARE-clinical at each in-person visit or telephone appointment if we may contact them later to share information about CARE-qualitative. We will reach out to women who agree to be contacted by phone, briefly explain the nature of CARE-qualitative, and schedule IDIs with willing and eligible individuals. Women in the OC/DMPA arm of CARE-clinical who opted not to try Annovera will be read a similar script at baseline and 4 weeks and undergo a similar procedure to schedule IDIs between weeks 4-8.

Annovera-using CARE-qualitative participants who express willingness to participate in IDIs will be also asked if they are comfortable for study staff to contact their male partner for a potential interview. For such women who agree and provide contact information for male partners, we will contact their male partners by phone, briefly explain the nature of the study, determine their willingness to participate, and schedule an interview if they are willing and eligible. Interviewers will not contact male partners of women using Annovera who are not comfortable with those partners being contacted. Interviewers will also not contact male partners of women who are not interested in participating in IDIs.

Finally, we will approach health care staff who provide Annovera as part of this study, explain the nature of the research and request an interview.

*2.4.2 Informed consent*

Potential CARE-clinical participants will meet with GCP-trained study staff (study site PI or designee) in a private space at the clinic to be informed about the study before any screening visit activities occur. If the participant cannot read, the participant will identify an impartial witness to attend the entire informed consent process. Signed informed consents will become part of the participant’s study record. Informed consent form templates for CARE-clinical are provided in Appendix 1a-b.

Informed consent for CARE-qualitative will be obtained on the day scheduled for interview by interviewers with training and experience in qualitative data collection. Written informed consent will be obtained for those who prefer in-person IDIs, and these individuals will have an opportunity to choose whether they prefer to read the informed consent form themselves or to have the interviewer read it to them. For individuals who prefer doing IDIs by phone, interviewers will read the informed consent form and ask participants to verbally indicate their willingness to participate. If a participant cannot read, an impartial witness will attend the informed consent process, except in the case of IDIs with health care providers, who are all literate and may not need the presence of an impartial witness. Model informed consent forms for CARE-qualitative are provided in Appendix 2a-c.

For both CARE-clinical and CARE-qualitative, informed consent activities will be conducted in the national language (Kiswahili in Kenya and Shona in Zimbabwe) or English, depending on the participant’s preference. A copy of the signed informed consent forms will be offered to participants to take home but they may choose not to do so if they fear loss of confidentiality. Informed consent forms for both CARE-clinical and CARE-qualitative will address various topics, including the aims of the study and methods to be used, institutional affiliations of the researchers, anticipated benefits and potential risks of study participation, duration of the study, compensation for study-related activities, discomfort that study participation may entail, the right to abstain from study participation or to withdraw from it at any time without reprisal, measures to ensure the confidentiality of information provided, planned uses for study data, and contact details for the investigators and national authorities. The CARE-clinical informed consent forms will also note that all Annovera rings (which includes used, unused, damaged, and expired rings) will be collected and shipped to a laboratory in the United States, where a subset of rings will undergo analysis on the remaining amount of active pharmaceutical ingredients in the ring at the end of its use.

*2.4.3 Inclusion and exclusion criteria*

Inclusion and exclusion criteria for both CARE-clinical and CARE qualitative are detailed in Table 1. In brief, CARE-clinical eligible participants are women aged 18-24 (in Zimbabwe) and 18-45 (in Kenya) at screening, who are at risk of pregnancy and seek to start a new contraceptive method, and who can safely use combined hormonal contraception and vaginal products. CARE-qualitative eligible participants are (1) women enrolled in either arm of CARE-clinical, or (2) male partners of women enrolled in the Annovera arm of CARE-clinical, or (3) health care providers providing Annovera at clinics participating in CARE-clinical. Potential CARE-clinical participants will be screened by nurses/clinicians based on medical history, clinical laboratory tests, and if required, a physical examination.

**Table 1.** CARE study inclusion and exclusion criteria.

	Inclusion criteria	Exclusion criteria
**CARE-clinical **	All female participants must meet the following criteria to be eligible for inclusion: A. Ages 18 through 24 (Zimbabwe) and 18 through 45 (Kenya) years old (inclusive) at screening, verified per site-specific SOPs B. Fluent in spoken Shona (Zimbabwe) or Swahili (Kenya) and/or English C. At risk for pregnancy (post-menarche, no prior sterilization, hysterectomy, oophorectomy, or menopause, per self-report) D. Contraceptive intentions: a. for potential Annovera users, desiring to initiate a new contraceptive method or change from one currently being used; or b. for non-Annovera users, has initiated or changed to OC or DMPA use at enrollment E. At least 12 weeks since last DMPA injection (per review of health record) if ever previously used DMPA F. Sexually active, defined as having had penile-vaginal intercourse with a man within the three months before screening G. Not pregnant at screening or enrollment (per negative pregnancy test) H. Not trying to become pregnant or intending to become pregnant within the next year I. No plans to move away from the study site in next 12 months J. Able and willing to provide informed consent K. Able and willing to provide adequate locator information L. In the opinion of the Site PI or designee, able and willing to comply with the protocol and all study procedures	Individuals who meet any of the following criteria (per self-report except where indicated) will be excluded from the study: A. BMI>29 (per exam at screening) B. Known hypersensitivity to or prior complications with estrogens, progestins, silicone or any of the other components of Annovera C. Not eligible for combined hormonal contraception; this includes an assessment of the following: a. Breastfeeding b. Less than 6 weeks (≤42 days) postpartum c. For women 35 and older, currently smokes cigarettes d. History of deep vein thrombosis or pulmonary embolism e. Prolonged immobilization or anticipating major surgery in the next year f. Thrombogenic valvular or thrombogenic rhythm diseases of the heart g. Inherited or acquired hyper-coagulopathies h. Current or history of cerebrovascular disease, coronary artery disease, or ischemic heart disease i. Uncontrolled hypertension or hypertension with vascular disease (self-report and clinic assessment) j. Systemic lupus erythematosus with positive or unknown antiphospholipid antibodies k. Migraines with aura l. For women over 35 years old, migraines without aura (applicable to Kenya) m. Current breast cancer or within 5 years of past breast cancer n. Diabetes for > 20 years and/or with nephropathy, retinopathy or neuropathy o. Symptomatic gall bladder disease p. Liver tumor, acute hepatitis or severe cirrhosis q. Undiagnosed abnormal vaginal bleeding r. Known condition where steroid hormones are contraindicated D. Use of medications that are contraindicated or believed to have a drug-drug interaction with Annovera E. Current abnormal vaginal discharge, genital tract lesions, or active pelvic infections (for woman in whom the clinician suspects a genitourinary tract infection, treatment should be offered and the woman can enroll following resolution of symptoms) F. History of genital tract surgery (including cervical polypectomy, dilatation and curettage, hysteroscopy, or laparoscopy) in the three months prior to the screening visit G. History of toxic shock syndrome H. Recurrent vulvovaginal candidiasis (defined as more than 3 episodes in the preceding year) I. Current use of vaginal rings (such as Dapivirine ring) or any other instance, as per clinician judgement, where using Annovera along with other vaginally inserted products is not advised J. Any other condition the clinician feels would jeopardize the health and well-being of the participant K. Previous enrollment in the trial as either an Annovera user or non-user
**CARE-qualitative **	**Women in CARE-clinical ** A. Enrolled in CARE-clinical. B. Able and willing to provide informed consent ** Male partners of women using Annovera in CARE-clinical ** A. Male partner of woman enrolled in the Annovera arm of CARE-clinical. B. Able and willing to provide informed consent ** Health care providers providing Annovera in CARE** A. Male or female individuals providing contraceptive services, and who have provided Annovera, at clinics participating in CARE-clinical B. Able and willing to provide informed consent	**Women in CARE-clinical ** A. Not enrolled in CARE-clinical. ** Male partners of women using Annovera in CARE-clinical ** A. Male partner for whom female partner did not provide permission to study staff for their male partner to be contacted. ** Health care providers providing Annovera in CARE** A. Individuals at clinics who participated in CARE, but who have not themselves provided Annovera.

*2.4.4 Screening and enrollment*

CARE-clinical: In CARE-clinical, women seeking to newly initiate or switch to a new method of contraception at the project sites will be provided with information about all contraceptive options typically available at that clinic, in addition to Annovera. They will freely choose the method that best fits their preferences, and in this way, will self-select their potential study arm (Annovera or OC/DMPA), and will proceed according to the schematic detailed in Figure 1. Those who express a preference to try Annovera and a subset of those who choose to try OCs or DMPA will be provided with information about the CARE study. If interested, they will undergo informed consent. Those who provide written informed consent will be assigned a study identification number, asked to provide contact information, and screened for eligibility by a study clinician, according to screening visit procedures in Table 2. Eligibility screening will involve assessing sociodemographic information; obtaining medical and menstrual history; reviewing concomitant medications; collecting height, weight, and blood pressure; confirming a negative urine pregnancy test; and (for women who choose to try Annovera, at minimum) a pelvic examination and/or targeted physical examination, if clinically indicated. Except for contraindicated medications [ 1], participants may use other medications during the study, and these will be recorded in case report forms. Use of items such as tampons, menstrual cups, and condoms are also permitted. Individuals who pass eligibility screening will receive the equivalent of approximately $10 USD to compensate them for their time and will be enrolled into CARE-clinical. At any point in time, enrolled participants may discontinue use of their contraceptive method (and thus their participation in CARE-clinical) but may not change the arm into which they were enrolled.

**Table 2.** CARE-clinical schedule of enrollment, interventions, and assessments.

	Appointment 1	Appointments 2-5	Appointment 6
	In-person Screening and Enrollment Appointment	Remote Appointments at Weeks 4, 12, 24, & 36 of method use	In-person End of Study Appointment or at discontinuation before 13 cycles of use
Procedure	ANNOVERA USERS: Screening & enrollment done on same day or enrollment procedures done within 30 days of screening visit	±1 week for Appointment 2±2 weeks for Appointment 3-5	Weeks 50-52 or at early discontinuation
Informed consent	X		
Assign participant study identification number (PID)	X		
Record/confirm locator information	X ^a^	X	X
Socio-demographics	X		
Medical history and menstrual history	X		
Review/record concomitant medications	X ^a^	X	X
Height	X		
Weight and BMI calculation	X ^a^		
Blood pressure	X ^a^		A
Pregnancy test	X ^a^		X
Pelvic examination and/or targeted physical examination if clinically indicated	[A]		[A]
Confirm eligibility	X ^a^		
Questionnaire	X ^b^	X	X
Protocol adherence & HIV/STI risk reduction counseling	X ^b^	X	X
Provide study product/participant insert in clinic	A ^b^		
Enquire about adverse events		A	A
Collect used Annovera rings			A

A: Annovera users only.

[A]: To be done for Annovera users only if clinically indicated.

^a^ If enrollment appointment is not on the same day as the screening appointment, procedure is to be repeated during the enrollment appointment.

^b^ Enrollment appointment procedure; to be done following confirmation of eligibility.

Enrollment procedures are slightly different by arm ( Figure 1). Women who express interest in trying Annovera after contraceptive counseling will be informed that this is only possible for those who enroll in CARE-clinical, since Annovera is not registered in their country. For Annovera-interested women willing to hear more about the study, staff will provide this information, obtain consent from willing individuals, and screen for eligibility. If an Annovera-interested woman is not interested in or eligible for CARE-clinical study participation, clinic staff will counsel her on other available contraceptive options. Study staff will explain to consented, eligible individuals how to use Annovera, provide the Annovera ring and a carrying case, have the participant insert Annovera, assess for discomfort, and enroll her into CARE-clinical. Participants contraindicated for Annovera use due to vaginal conditions identified during ring insertion and who are withdrawn from the study will be replaced. Participants eligible for Annovera can be enrolled within 30 days of the screening visit, but if more than 30 days elapse between screening and enrollment, screening procedures will be repeated. This will occur only once; if a potential participant does not enroll after the second screening visit attempt, she will be excluded from participation.

In contrast, participation in the CARE study is not required for women who wish to try OCs or DMPA, methods which have long been available in these study clinics. To minimize disruption to clinic flow, women who choose to try OCs or DMPA and are medically eligible will be provided their chosen method prior to being presented with information about the study. Only a subset of women who choose to try OCs or DMPA will be invited to learn more about the CARE study, and, if interested, will undergo informed consent and screening ( Table 2).

The number of participants enrolled in the OC/DMPA arm at each study site will not be permitted to exceed the number of participants enrolled in the Annovera arm. For instance, if 12 participants have enrolled in the Annovera arm, all women initiating either OCs or DMPA will be invited to join CARE-clinical until both arms have 12 enrolled participants. Conversely, if both arms contain enrolled 12 participants, we will not invite additional individuals choosing to try OC/DMPA to learn more about CARE until additional participants enroll in the Annovera arm. If Annovera arm enrollment unexpectedly outpaces OC/DMPA arm enrollment by more than 10 participants at a study site, we will review enrollment data to determine whether to pause Annovera enrollment until both arms have the same number of enrolled participants. We will not aim to match enrollers by any demographic factors.

CARE-qualitative : If the number of women willing to participate in the qualitative study exceeds the required sample size (see section 2.7), we will select participants from this pool based on ensuring diversity in age, education, and marital status. Recruitment of male partners will depend on women in CARE-clinical who are comfortable with their male partners being contacted, and on whether those male partners provide consent. Among such individuals, we will invite all male partners until the desired sample size is reached. The number of eligible health care staff to recruit is pre-determined, based on their role providing Annovera in the participating clinics. As there are few staff who will provide Annovera, we will recruit all who grant consent to participate.

*2.4.5 Study appointments*

CARE-clinical : As described in Table 2, at Appointment 1 (baseline visit), enrolled participants will receive counseling related to protocol adherence and to HIV/STI risk reduction and will be offered male and/or female condoms. Study staff will then administer a baseline questionnaire, anticipated to take approximately 1-1.5 hours, and will schedule follow-up appointments. At the end of this visit, participants will receive the equivalent of approximately $10 USD (in addition to the compensation received for screening procedures) to compensate them for their time.

Appointments 2-5 will be conducted remotely via telephone contacts to emulate the experience of not being required to return to the clinic during one year of Annovera use. If a participant does not own their own phone, a study staff member will visit the participant at a location of their choice and provide them with a phone to use for the duration of the appointment. Calls will occur at the following time points in relation to the date of enrollment: Appointment 2 = 4 weeks ± 1 week; Appointment 3 = 12 weeks ± 2 weeks; Appointment 4 = 24 weeks ± 2 weeks; Appointment 5 = 36 weeks ± 2 weeks. These calls will include procedures detailed in Table 2, including administration of a survey anticipated to take approximately 20-30 minutes. At the end of each appointment, participants will receive the equivalent of approximately $5 USD to compensate them for their time, which may be delivered via a mobile phone-based money transfer if the participant wishes.

Appointment 6 will occur in-person at the study site between weeks 50-52 after enrollment. This visit will include procedures detailed in Table 2, including administration of a survey anticipated to take approximately 1 hour. At this visit, women in the Annovera arm will be asked to return their ring to the clinic. Study staff will discuss options for subsequent contraceptive care during this visit with all participants. At the end of this visit, participants will receive the equivalent of approximately $10 USD per appointment to compensate them for their time.

We anticipate that a proportion of CARE-clinical participants will discontinue before a full year of use and respect the right of participants to discontinue using their contraceptive method and to discontinue study participation. Participants who stop using their contraceptive method prior to 52 weeks of use for any reason, including pregnancy, or who wish to end their participation in the study, will be discontinued from the study. They will be asked to complete the procedures outlined for the End of Study appointment ( Table 2), which will include completing an early termination survey. At the end of this visit, these individuals will receive the equivalent of approximately $10 USD to compensate them for their time. Annovera users who discontinue Annovera use due to an adverse event (AE) will continue to be followed until resolution or stabilization of the AE is documented.

CARE-clinical study participants who become pregnant will be discontinued from the study and referred for follow-up per standard of care at the clinic. Any pregnancies to Annovera users will be followed until outcome (i.e., delivery or termination of pregnancy (spontaneous or induced)).

A CARE-clinical study participant will be considered lost to follow-up if she is repeatedly unresponsive to a minimum of three attempted contacts, each on different days, using various methods of communication (e.g., cell phone, home visit, email) for completion of study appointments.

At End of Study visits, Annovera-using CARE-clinical participants will be asked to return their ring for discoloration assessment. A subset of rings will be assayed for residual drug levels (segesterone acetate and ethinyl estradiol).

CARE-qualitative : Serial cross-sectional IDIs will be conducted with women using or who have discontinued using Annovera at 4, 24 and 52 weeks following method initiation. Interviews will be conducted in-person or over the phone (as per participant’s preference) using a guide. We anticipate that the process of obtaining consent and conducting the interview will take approximately 1 – 1.5 hours.

Women in the OCs/DMPA arm will be interviewed at 4 to 8 weeks following method initiation and will be taken through similar consenting and interview procedures as Annovera users by CARE-qualitative study staff. Male partners of Annovera users will, on the other hand, be interviewed between 12 to 24 weeks following their female partner’s initiation of Annovera. Study staff will take these men through similar consenting and interview procedures as done with women in CARE-qualitative. Women participating in the interviews (in the Annovera or OCs/DMPA arm) at any of the time points and male partners of Annovera users will be compensated at the equivalent of approximately US $5-10 USD (with variation by country) for out-of-pocket expenses (e.g., traveling to and from the study site, their time and inconveniences as a result of participation).

Interviews with health care providers providing Annovera will be conducted when nearly all women continuing to use Annovera have completed the full 13 cycles. Interviews with healthcare providers will be in-person. Unlike other groups of CARE-qualitative participants, health care providers will not receive compensation for participating in the qualitative interviews.

*2.4.6 Study monitoring*

CARE-clinical study monitoring visits will consist of a site initiation visit, periodic monitoring visits, and a site close-out visit. During routine monitoring visits, the monitor will review study documents to verify compliance with the protocol and will review the source documents to confirm the accuracy of the data recorded on the case report forms (CRFs). We established a Data Monitoring Committee (DMC) consisting of three experts with expertise in contraception, epidemiology, and conduct of clinical trials, and who have no financial, scientific, or other conflict of interest with this study. The DMC (charter available on request) will review data approximately every six months, or on an *ad hoc* basis should unanticipated problems arise. They may provide recommendations about study termination or other modifications to the study.

For CARE-qualitative, participants may be contacted by the study sponsor to determine if they gave informed consent to participate in the research.

*2.4.7 Safety monitoring and adverse event reporting*

The Site PI or designee will monitor and record adverse events (AEs) and serious adverse events (SAEs) for Annovera and non-Annovera users throughout CARE-clinical. AEs and SAEs will be recorded in the appropriate case report forms (CRFs) for inclusion in the study database, with the Site PI or designee determining relatedness, severity, and seriousness. Participants who become pregnant will be terminated from the study and referred for antenatal services. The sites will make every effort to follow-up on all pregnancy outcomes and document in study CRFs.

Annovera and non-Annovera users will be instructed to contact the study site staff to report any AEs and SAEs they experience. At each in-person and remote study appointment, Annovera users will be asked about their general health (how they have been feeling) since their previous appointment. As needed, Annovera users will be examined if symptomatic during an in-person visit or be asked to come to the study site if the appointment is remote.

All SAEs and AEs that lead to study discontinuation will be reported to the relevant IRBs/ethics committees and drug regulatory bodies consistent with applicable regulations, the DMC, and as relevant, the Annovera license holders (in accordance with the approved safety data exchange agreements). AEs and other clinical events will be managed appropriately, in the facility that provided Annovera or by referral to a higher-level facility, if necessary.

Social harms of study participation may include negative consequences related to stigma, discrimination, confidentiality breaches, and psychological distress. Participants will be asked about social harms at study appointments and will be referred to counseling and other support services as needed. Social harm events will be recorded and reported to the relevant IRBs/ethics committees, as applicable. Physical harm related to study participation will be reported as a social harm and any resulting medical adverse consequences (e.g., pain, bruising) will be reported as an AE.

If AEs or social harms are identified during CARE-qualitative, the qualitative team will inform the clinical team as it could be that the participant might not have reported the AEs or social harms to the clinical team.

*2.4.8 Confidentiality*

We will make every effort to ensure the privacy and confidentiality of data collected from all participants screened and enrolled in the clinical or qualitative components of the CARE study. In-person interviews will be conducted in private locations, and telephonic interviews will occur in the location of the participant’s choosing after confirming with the participant that the environment ensured privacy. Access to identifiable information in the research data will be limited to research staff only. Participants’ identifiable study information will not be released without their written permission, except as necessary for review, monitoring, and/or auditing by representatives of the Population Council and its monitors, study staff and/or their representatives, national health ministries, site IRBs, and regulatory authorities. All information disclosed in reports, presentations, or publications will be anonymous. Any breaches in confidentiality will be reported to the relevant IRBs/ethics committees and regulatory authorities.

In CARE-clinical, consented participants will be assigned a unique Participant Identification (PID) Number; no names or other identifying information will be entered into REDCap. All data collected will be recorded using PIDs; no names or other identifying information will appear on any clinical forms, documents, specimens, or any other materials. Study staff will maintain a participant identifier log that includes a list of PIDs with associated names and contact information in a secure location with limited access. All identifiable information will be destroyed after the retention periods prescribed by local regulatory requirements. In CARE-qualitative, all collected data will be treated confidentially and participant names will not appear on the interview transcripts. Rather, pseudonyms will be used to identify participants and transcripts will be identified using PIDs generated by the CARE-clinical component. All data will be kept separately from any identifying information that might be captured in the consent forms. Transcripts will be anonymized and archived in a password-protected computer, and audio transcripts will be erased after the study report is finalized.

### 2.5 Primary and secondary outcomes

Primary and secondary outcomes are detailed in Table 3 for both CARE-clinical and CARE-qualitative, and also described in section 2.6.3.

**Table 3.** CARE study objectives.

CARE-CLINICAL			
	Objectives	Endpoints	Assessments
Primary objectives	To assess method satisfaction in Annovera users vs. OC/DMPA at 12 and 52 weeks of use.	Proportion of participants reporting they are “highly satisfied” or “satisfied” with their method.	Acceptability questionnaires administered during follow up appointments (includes question on satisfaction with method.)
	To compare method continuation between Annovera users and OC/DMPA users at 4, 12, 24, 36, and 52 weeks.	Kaplan-Meier survival curves for method continuation over time.	Self-report of continued method use, date of and reason for discontinuation.
	To assess Annovera uptake by the proportion of women at the site during the study period who initiated Annovera vs. OC/DMPA.	Proportion of women who initiate Annovera (or OCs/DMPA) among all women who newly initiated a method at the study site during the study period.	Review of clinic records documenting all women seeking contraception during study and method selected.
Secondary objectives	To assess various dimensions of acceptability of Annovera at 4, 12, 24, 36 and 52 weeks.	Aspects to be explored include: ease of use, experience and sensation, impacts on sexual activity, as well as use patterns, likes/dislikes, hypothetical cost considerations, comparison with other methods, and plans for future method use.	Acceptability questionnaires administered during follow-up appointments, and information from assays (to compare assay-derived use vs. self-reported use).
	To determine characteristics associated with willingness to try Annovera (vs. to use other contraceptive methods).	Profiles of women who try Annovera or another method.	Baseline questionnaires (Annovera users and OC/DMPA users).
	To determine characteristics associated with satisfaction with Annovera (vs. with dissatisfaction with Annovera).	Profiles of women who are satisfied with Annovera.	Follow-up questionnaires for Annovera users.
	To assess reasons for contraceptive method selection and discontinuation in Annovera users and users of other contraceptive methods.	Comparison of reasons for contraceptive method selection and discontinuation in Annovera users and non-users.	Baseline questionnaire and discontinuation survey.

CARE-QUALITATIVE	
Primary objectives	Assessments
To assess factors influencing acceptability and perceptions of women aged 18-45 years in Kenya and Zimbabwe regarding Annovera	IDIs with women in CARE-clinical
To assess the perceptions of husbands/partners of women using Annovera about the method	IDIs with male partners of women using Annovera in CARE-clinical
To assess the perceptions and provision practices of healthcare providers regarding Annovera	IDIs with health care workers who have provided Annovera
To assess factors influencing method choice among women who do not choose Annovera and instead choose OCs or DMPA	IDIs with women using OCs/DMPA in CARE-clinical

### 2.6 Data collection, management, and analysis

*2.6.1 Data collection*

In CARE-clinical, we will collect data via source documents, Case Report Forms (CRFs), quantitative surveys, and ring assays. We developed CRFs to capture information about enrollment-related data, medical and menstrual history, concomitant medications, pelvic and physical exams, pregnancy testing and notifications, ring dispensing and return, adverse events, social harms, missed appointments, protocol deviations, and study termination. Quantitative surveys will be administered by a study staff member, either in-person or telephonically, at each study appointment. To develop these questionnaires, we drew on Sekhon *et al.*’s contraceptive acceptability model ^27^ and Griffin *et al.*’s subsequent operationalization of that model, ^22^ and also used or adapted some questions from prior vaginal ring ^28– 31^ and other contraceptive acceptability studies. ^32, 33^ Our questionnaires (baseline, follow-up, and early discontinuation) are available in Appendix 3a-3c. We will also gather information from the rings that are returned to the United States after use for laboratory analysis. No human biological material will be used or stored.

For CARE-qualitative, we will conduct cross-sectional IDIs, captured using audio recorders. Study guides for CARE-qualitative were informed by similar studies on acceptability of contraceptive vaginal rings, ^34– 36^ and are available in Appendix 4a-d.

For both CARE-clinical and CARE-qualitative, interviews will be conducted in English or in the national language (Kiswahili in Kenya and Shona in Zimbabwe), according to the participant’s preference.

*2.6.2 Data management*

For CARE-clinical, study data will be collected and managed using Research Electronic Data Capture (REDCap), an electronic data collection tool on a validated server hosted at the Population Council. ^37, 38^ REDCap is a secure, web-based software platform designed to support data capture for research studies. Queries will be triggered during data entry or by study data managers during routine data reviews. Study sites will maintain source data and documents in secure locations with limited access for the entire period of study implementation and until after the retention periods prescribed by local regulatory requirements.

For CARE-qualitative, audio-recorded interviews will be transcribed and typed in Word by the interviewers. Interviews conducted in the national language (Kiswahili in Kenya and Shona in Zimbabwe) will be transcribed directly from the national language to English by interviewers. Transcripts will be submitted to the study investigators via a secure link accessible only to members of the research team. Audio recordings will be erased once the study reports are finalized.

In line with funding requirements, data underlying any CARE publications will be anonymized and made publicly available through online data repositories. If anonymity cannot be ensured through protective measures like de-identification, or if sharing could put participants at risk of re-identification, we will seek an exception from the funding agency.

*2.6.3 Data analysis*

For CARE-clinical, we will assess three primary objectives. First, we will calculate the proportion and 95% confidence interval for **Annovera uptake** as compared with uptake of OCs and DMPA (calculated as the number of women who initiated these methods among all women who newly initiated contraception or switched from another method at the study site during the study timeframe). We will measure the cumulative probabilities of **continuation** of Annovera as compared with continuation of OCs/DMPA over 52 weeks, based on Kaplan-Meier methods: plotting survival curves and calculating 95% confidence intervals around the cumulative probabilities of continuation. We will calculate **satisfaction** as the proportion of women who report being “satisfied” or “very satisfied” with Annovera versus with OCs/DMPA, based on the questionnaire administered at weeks 12 and 52. We will compare satisfaction by method and timepoint using mixed-effects logistic regression with subject and timepoint (week 12 or 52) as random effects and satisfaction as the dependent variable.

We will also assess four secondary objectives. First, we will assess the **acceptability** of Annovera using the IVR acceptability scale (IVR-AS) which assesses ease of use, experience with the ring/sensation and effects on sex and intimacy. Other aspects from the acceptability questionnaire will also be summarized including likes/dislikes, hypothetical cost considerations, comparison with other methods, adherence, and plans for future method use. Acceptability endpoints will be summarized by time point to assess patterns over time and to determine potential subsequent analyses. We will also summarize and compare baseline characteristics among participants **willing to try** Annovera versus those willing to try OCs/DMPA using chi-square or t-tests. Similarly, we will compare baseline characteristics among participants in the Annovera arm who reported that they were **satisfied** or very satisfied (vs. dissatisfied or very dissatisfied) with the ring. Depending on patterns of satisfaction responses over time, we will either do this comparison using mixed-effects models to account for repeated responses, or will categorize participants by their pattern of responses (e.g. always satisfied, initially dissatisfied-then satisfied, etc.) We will compare reasons for **contraceptive method selection** and reasons for **discontinuation** between participants in the Annovera and OC/DMPA arms using chi-square tests.

We will assess all returned vaginal rings to evaluate the extent of discoloration, according to established visual assessment methods. ^39^ We will also assay approximately 50-60 returned rings for residual drug levels (segesterone acetate and ethinyl estradiol) from participants who discontinued at various timepoints. Specifically, we will aim to assay rings from approximately 6 participants (3 per site) discontinuing by each of weeks 4, 12, 24, and 36 and from approximately 30 participants (approximately 15 per site) completing the study (a total of 50-60 assayed rings out of the expected 200 used in the study). We will aim for the number of rings assayed at each time point to be similar across both study sites. We will compare residual drug (NES, EE) content of these rings against self-reported days of ring use.

All enrolled participants will be included in all analyses where they contributed data. Select analyses will also be done for the following subgroups: country (Kenya, Zimbabwe), age group (18-24, 25-35, 36-49). Missing data will be treated as missing and not imputed. A statistical analysis plan, to be completed and approved prior to data lock, will provide further detail on analysis specifications.

For CARE-qualitative, we will employ a thematic content analysis approach. This will entail coding the data, developing a list of emerging themes, categorizing themes within a hierarchical framework of main and sub-themes, looking for patterns and associations between themes, and comparing within and between participant groups. CARE-qualitative study investigators will work with two coders (per country) to develop a codebook based on the initial interviews. The coders will then use the codebook to code the remaining transcripts. Any disagreements in coding of transcripts between the two coders will be resolved through discussions between the study investigators and the coders. Insights generated from the qualitative data will elucidate some of the patterns observed in the clinical data, for example, on factors that could explain those patterns.

### 2.7 Sample size calculations

As CARE-clinical is an acceptability study, we did not develop formal statistical hypotheses nor base sample size on statistical considerations. With 200 women in each group, the standard error around a point estimate (e.g., satisfaction) would be at most 7%.

For CARE-qualitative we purposively determined sample sizes based on the minimum number of interviews that would yield saturation while ensuring representation from those using the method at the time of interview and those who stopped, in addition to considering resource constraints. Research suggests that saturation is achieved with the first 12 interviews while basic elements of meta-themes are present in the first six interviews. ^40^ From the Annovera arm of CARE-clinical, we will recruit up to 30 women in Kenya and 18 in Zimbabwe), and up to 6 male partners of these women in each study site. From the OC/DMPA arm of CARE-clinical, we will recruit up to 6 women in each study site. A total of 6 health care staff (about 3 in each country) providing Annovera in clinics participating in the research will be targeted for inclusion in the study; this was determined based on the number of staff offering contraceptive services in participating clinics as part of this study.

### 2.8 Ethics and dissemination

*2.8.1 Ethics*

For CARE-clinical, the protocol (V.6.0, 10 May 2024), informed consent forms, data collection instruments, counseling materials, and recruitment materials were approved by the Institutional Review Board of the Population Council (New York, New York, USA) on 04 August 2024 number 984. In Kenya, the protocol, informed consent forms, data collection instruments (in English and Swahili), counseling materials, and recruitment materials were approved by the Scientific and Ethics Review Unit (SERU) on 09 July 2024 number 4810 at the Kenya Medical Research Institute (KEMRI). In Zimbabwe the protocol, informed consent forms, data collection instruments (in English and Shona), counseling materials, and recruitment materials were approved by the Medical Research Council of Zimbabwe (MRCZ) on 15 July 2024 number MRCZ/A/3002. We are in the process of receiving approval for the Protocol from the Mèdicines Sans Frontières Ethics Review Board (MSF ERB).

The study will be conducted in accordance with the US Code of Federal Regulations, the International Conference for Harmonisation of Technical Requirements for Pharmaceuticals for Human Use Guideline for Good Clinical Practice E6 (R2), Guidelines for Good Clinical Trial Practice in Zimbabwe (dated 30 August 2024) and local standard operating procedures at each site. The study is registered on the Pan African Clinical Trials Registry (202312652733486). The WHO trial registration data set is available in Table 4.

**Table 4.** Trial registration data for CARE-clinical.

Data category	Information
Primary registry and trial identifying number	PACTR 202312652733486
Date of registration in primary registry	December 14, 2023
Secondary identifying numbers	N/A
Source(s) of monetary or material support	Bill and Melinda Gates Foundation
Primary sponsor	Population Council
Secondary sponsor(s)	N/A
Contact for public queries	Dr. Chelsea Polis
Contact for scientific queries	Dr. Chelsea Polis
Public title	Contraceptive Acceptability REsearch (CARE)
Scientific title	Assessing acceptability of Annovera contraceptive vaginal ring use (compared to other contraceptive method use) among women seeking family planning services
Countries of recruitment	Kenya, Zimbabwe
Health condition(s) or problem(s) studied	Contraceptive acceptability
Intervention(s)	Annovera vaginal ring versus oral contraceptive pills or contraceptive injections
Key inclusion and exclusion criteria	See registration
Study type	Comparative non-randomized acceptability study
Date of first enrollment	16 October 2024
Target sample size	400
Recruitment status	Recruiting
Primary outcome(s)	Method uptake, method continuation, and method satisfaction
Key secondary outcomes	Various domains of acceptability, characteristics associated with willingness to try Annovera, characteristics associated with satisfaction with Annovera, reasons for contraceptive method selection and discontinuation.

For CARE-qualitative, the protocol and amendments (dated June 22, 2023), informed consent forms, and study guides were approved by the Institutional Review Board of the Population Council (New York, New York, USA) on 25 October 2023 number 1026. In Kenya, the protocol and amendments (Protocol ESRC P1543/2023) was approved by AMREF Ethics and Scientific Review Committee on 30 November 2023. In Zimbabwe, the protocol was approved by the MSF ERB on 29 February 2024 number 2372, and at the time of submission of this paper, additional approvals are ongoing.

*2.8.2 Conflict of interests*

Annovera was developed by the Population Council and is currently licensed by Mayne Pharma for the US market.

*2.8.3 Access to data*

A data sharing agreement between the Population Council, MSF, KEMRI, and the Harare Health Research Consortium (HHRC) will be signed for CARE data, stating that the parties may share study datasets and confidential information with each other pursuant to the terms of the agreement, and solely for the purposes of carrying out the study objectives. Any other use or transfer to any third party will require prior and written approval of all other parties, and as required by applicable laws and the competent authorities in each country. As described above, and in line with funding requirements, data underlying any CARE publications will be anonymized and made publicly available through online data repositories, subject to any applicable ethical, legal, or regulatory requirements or restrictions.

*2.8.4 Post-trial care*

Annovera users in CARE-clinical who wish to continue using Annovera after the study ends will be offered Annovera free of charge for a period of three years post-study. Prior to the end of the study, we will develop a plan to ensure this three-year post-study access via an appropriate participant program (e.g., an expanded protocol), based on feasibility assessment and feedback from research centers. We will also ensure compliance with country-specific regulations and laws.

If a CARE-clinical participant needs treatment because of an injury sustained through study participation, important health services and short-term medical assistance and treatment will be available for free immediately as deemed by the study doctors. We will not offer monetary compensation for any services, but healthcare discussion and appropriate referrals to other healthcare services will be available.

*2.8.5 Dissemination*

The CARE study team will periodically update the study communities and Community Advisory Boards about progress during study implementation. On completion, results will be presented locally to study participants, community advisory boards, and other local stakeholders, as well as shared at national and international meetings and conferences. Manuscripts will be submitted to peer-reviewed journals and made available via Open Access terms, as per funder policy. An authorship committee including representatives from each entity participating in study implementation will be responsible for collaboratively ensuring equitable collaboration and ethical authorship practices, including adherence to International Committee of Medical Journal Editors (ICMJE) guidelines.

## 3.0 Discussion/Conclusions

The CARE study will shed light on the uptake, continuation, and acceptability of contraceptive vaginal rings in ‘real-world’ contraceptive service settings in two sub-Saharan African countries. Findings will be based on actual ring use and contextualized via comparison to two other commonly available contraceptive methods. As vaginal ring delivery systems are currently being considered for multiple reproductive health indications in addition to contraception, this study will fill key knowledge gaps and equip decision-makers with critical information needed to inform future investments in reproductive health.

## Data availability statement

### Underlying data

No data are associated with this article.

### Reporting guidelines

SPIRIT checklist for “A comparison of acceptability of contraceptive vaginal rings, pills, and injectables among cisgender women in Kenya and Zimbabwe: protocol for a mixed-methods study”, https://doi.org/10.7910/DVN/ZFJZ2D. ^41^

Also at the above location are the following Appendices:

• Appendix 1a: Model informed consent form for Annovera users (Clinical)
• Appendix 1b: Model informed consent form for non-Annovera users (Clinical)
• Appendix 2a: Model informed consent form for women (Qualitative)
• Appendix 2b: Model informed consent form for male partners of Annovera users (Qualitative)
• Appendix 2c: Model Informed consent form for health care staff providing Annovera
• Appendix 3a: Baseline questionnaire (Clinical)
• Appendix 3b: Follow up questionnaire (Clinical)
• Appendix 3c: Early discontinuation questionnaire (Clinical)
• Appendix 4a: Model in-depth interview guide for Annovera users (Qualitative)
• Appendix 4b: Model in-depth interview guide for non-Annovera users (Qualitative)
• Appendix 4c: Model in-depth interview guide for male partners of Annovera users (Qualitative)
• Appendix 4d: Model in-depth interview guide for health care staff providing Annovera (Qualitative)

Data are available under the terms of the Creative Commons Zero “No rights reserved” data waiver (CC0 1.0 Public domain dedication).

### Role of study sponsor and funders

The study sponsor (Population Council, New York, NY) was involved in study design and will be involved in collection, management, analysis, and interpretation of data, writing of reports, and the decision to submit reports for publication. The funder reviewed plans for study design and will have no role in data collection and analysis, decision to publish, or preparation of the manuscript. The conclusions and opinions expressed in this work are those of the authors alone and shall not be attributed to the Foundation. Under the grant conditions of the Foundation, a Creative Commons Attribution 4.0 Generic License has already been assigned to the Author Accepted Manuscript version that might arise from this submission. Please note that works submitted as a preprint have not undergone a peer review process.

**Sponsor**: Population Council, Center for Biomedical Research, New York, NY.

## Footnotes

^1^
Antibiotics: Rifamycins (Rifambutin or Rifampicin). Antifungal: Griseofulvin. Anticonvulsants: Carbamazepine, Emosuximide, Phenorbabitone, Phenytoin, Primidone, Topiramate, Oxcarbazepine, Lamotrigine. Antiretrovirals: Fosamprenavir, Efavirenz. Hepatitis C Treatment: Ledipasvir/sofosbuvir, ombitasvir/paritaprevir/ritonavir, with or without dasabuvir. Oil-based vaginal suppositories. Oil-based (including silicone-based) vaginal lubricants. St. John’s Wort. Investigational therapies for any medical condition.
